# Heat Shock Protein A6, a Novel HSP70, Is Induced During Enterovirus A71 Infection to Facilitate Internal Ribosomal Entry Site-Mediated Translation

**DOI:** 10.3389/fmicb.2021.664955

**Published:** 2021-05-07

**Authors:** Yu-Siang Su, Lih-Hwa Hwang, Chi-Ju Chen

**Affiliations:** ^1^Institute of Microbiology and Immunology, National Yang Ming Chiao Tung University, Taipei, Taiwan; ^2^Institute of Microbiology and Immunology, National Yang-Ming University, Taipei, Taiwan

**Keywords:** enterovirus A71 (EV-A71), HSPA6, internal ribosomal entry site, viral IRES, induced HSP70

## Abstract

Enterovirus A71 (EV-A71) is a human pathogen causing hand, foot, and mouth disease (HFMD) in children. Its infection can lead to severe neurological diseases or even death in some cases. While being produced in a large quantity during infection, viral proteins often require the assistance from cellular chaperones for proper folding. In this study, we found that heat shock protein A6 (HSPA6), whose function in viral life cycle is scarcely studied, was induced and functioned as a positive regulator for EV-A71 infection. Depletion of HSPA6 led to the reductions of EV-A71 viral proteins, viral RNA and virions as a result of the downregulation of internal ribosomal entry site (IRES)-mediated translation. Unlike other HSP70 isoforms such as HSPA1, HSPA8, and HSPA9, which regulate all phases of the EV-A71 life, HSPA6 was required for the IRES-mediated translation only. Unexpectedly, the importance of HSPA6 in the IRES activity could be observed in the absence of viral proteins, suggesting that HSPA6 facilitated IRES activity through cellular factor(s) instead of viral proteins. Intriguingly, the knockdown of HSPA6 also caused the reduction of luciferase activity driven by the IRES from coxsackievirus A16, echovirus 9, encephalomyocarditis virus, or hepatitis C virus, supporting that HSPA6 may assist the function of a cellular protein generally required for viral IRES activities.

## Introduction

The enterovirus A71 (EV-A71) belongs to the genus *Enterovirus* in the family *Picornaviridae*. EV-A71 infection often manifests as hand, foot, and mouth disease (HFMD), but occasionally leads to life-threatening neurological complications such as aseptic meningitis, brain stem encephalitis or acute flaccid paralysis (McMinn, 2002). EV-A71 is currently considered as the most neurotropic enterovirus and a public health issue worldwide (Yi et al., 2017; Baggen et al., 2018).

The RNA genome of EV-A71 is approximate 7.4 kilobases and encodes a single polyprotein. Following the steps of attaching and penetrating *via* receptor-mediated endocytosis, viral RNA (vRNA) of EV-A71 is released into the cytoplasm by an endosome-dependent uncoating process (Hussain et al., 2011; Lin et al., 2013). The genomic RNA, also functioning as the mRNA, is immediately translated into a polyprotein, which is then processed into 10 different viral proteins including viral RNA polymerase 3D^*pol*^ by viral proteases 2A^*pro*^, 3C^*pro*^, and 3CD^*pro*^. The 3D^*pol*^ directs the synthesis of both genomic and antigenomic RNA in a membrane-associated replication complex containing viral proteins or their precursors including 2BC, 2B, 2C, 3A, and 3CD^*pro*^ as well as host proteins (Ilnytska et al., 2013; Paul and Wimmer, 2015; van der Schaar et al., 2016). The newly synthesized genomic RNA is then packaged into viral particles followed by a maturation process (Basavappa et al., 1994). Most assembled virions are released upon cell lysis as a result of the viral protein-induced apoptosis (Cong et al., 2016; Li et al., 2017); on the other hand, a small portion of the virions can be released *via* membrane-bound vesicles in a non-lytic manner (Feng et al., 2013; Bird et al., 2014; Robinson et al., 2014; Chen et al., 2015; Too et al., 2016).

EV-A71 translation is mediated by the internal ribosome entry site (IRES) located at a highly structured 5′ untranslated region of the viral genome. The IRES-mediated translation allows viral proteins to be synthesized while host cap-dependent translation is shut off during viral infection (Kuyumcu-Martinez et al., 2002; Thompson and Sarnow, 2003; Kuyumcu-Martinez et al., 2004; de Breyne et al., 2008). Nevertheless, the IRES-mediated translation requires canonical translational initiation factors such as eIF4A, eIF4B, eIF3, eIF2, and eIF1A (Sweeney et al., 2014), as well as the 2A^*pro*^-cleaved eIF4G (Kräusslich et al., 1987; Lamphear et al., 1993; Sommergruber et al., 1994; Haghighat et al., 1996; Gradi et al., 1998). In addition to canonical translational factors, several cellular IRES *trans*-acting factors (ITAFs) modulate IRES activity [reviewed in Lozano and Martínez-Salas (2015)]. Up to date ITAFs known to promote the activity of EV-A71 IRES include heterogeneous nuclear ribonucleoprotein A1 (hnRNP A1) (Lin et al., 2009; Levengood et al., 2013), poly (rC)-binding proteins 1 and 2 (PCBP1/2) (Choi et al., 2004; Luo et al., 2014), polypyrimidine tract-binding protein 1 (PTBP1) (Xi et al., 2019), HuR, Ago2 (Lin et al., 2015), the 68-kDa Src-associated protein in mitosis (Sam68) (Zhang et al., 2015), far-upstream element-binding protein 1 (FBP1) (Hung et al., 2016), and DDX3X RNA helicase reported by us (Su et al., 2018).

When cells are exposed to stress, heat shock proteins (HSPs) are synthesized at increased level to maintain cellular homeostasis. HSP70 is a subfamily of HSP superfamily with approximate 70 kDa molecular weight, which accounts for the majority of molecular chaperones in cells (Saibil, 2013). There are at least 14 human HSP70 proteins that differ in aspects like expression level, subcellular localization, and inducibility to stress (Stricher et al., 2013). They can be classified into “stress-induced,” e.g., HSPA1 (aka HSP72) and HSPA6 (aka HSP70B’), and “constitutively expressed,” e.g., endoplasmic reticulum (ER)-residing HSPA5 (aka GRP78), HSPA8 (aka HSC70), and mitochondrial-residing HSPA9 (aka GRP75). HSP70 proteins, whose binding to client proteins is regulated by ATP hydrolysis, generally do not work alone but cooperate with HSP40 cochaperones (Mayer, 2013). Other than helping cells to cope with stress, HSP70s also participate positively in diverse steps of the virus replication cycle. For instances, HSPA1 facilitates replication complex formation for hepatitis C virus (HCV) (Chen et al., 2010). HSPA5 assists entry and replication for Japanese encephalitis virus (JEV) (Nain et al., 2017) and coxsackievirus A9 (CA-V9) (Triantafilou et al., 2002). HSPA8 has roles in divergent steps during the infection of Dengue virus, Rotavirus or JEV (Taguwa et al., 2015; Zárate et al., 2003; Chuang et al., 2015). It also regulates EV-A71 IRES-mediated translation by interacting with 2A^*pro*^ and promoting eIF4G cleavage (Dong et al., 2018). In addition, we reported that HSPA1, HSPA8 and HSPA9 regulate multiple steps of the EV-A71 life cycle including IRES-mediated translation (Su et al., 2020). These are just few examples to demonstrate the importance of HSP70s in various steps of virus infection processes. In addition to HSP70s, HSP27 facilitates EV-A71 IRES activity by enhancing 2A^*pro*^-dependent eIF4G cleavage, a function similar to HSPA8, and hnRNP A1 cytoplasmic translocation (Dan et al., 2019).

Both HSPA1 and HSPA6 (sharing 85% sequence identity) (Daugaard et al., 2007; Stricher et al., 2013) are stress-induced HSP70s. While the functions of HSPA1 in virus replication cycle have been discussed widely [reviewed in Santoro et al. (2009)], there is little information regarding the function of HSPA6 in viral life cycle. HSPA6 is strictly inducible, having low or non-detectable expression levels in most cells (Noonan et al., 2007a, b), and may share overlapping functions with HSPA1 in response to cellular stresses (Daugaard et al., 2007; Noonan et al., 2007a). The only report showing HSPA6 may have any role in a given virus is its association with the 3′UTR of HCV genome (Harris et al., 2006); however, the meaning of this association is not clear.

In this study, we found that HSPA6 protein was induced during EV-A71 infection. Depletion of HSPA6 led to the reductions of viral proteins, viral RNA, and virion production, indicating that HSPA6 had a positive role in the EV-A71 life cycle. We investigated at which step HSPA6 was involved during EV-A71 infection and found that it was only required for the IRES-mediated translation. Unexpectedly, the negative consequence of HSPA6 depletion in the IRES-mediated translation could be observed in the absence of viral proteins, suggesting that HSPA6 acted on cellular factors to facilitate IRES activity. Alternatively, it may affect viral IRES activity by its mRNA binding activity (Harris et al., 2006). Intriguingly, knockdown of HSPA6 also affected the translation directed by the IRES from coxsackievirus A16 (CV-A16), echovirus 9 virus (echo 9), encephalomyocarditis virus (EMCV), or hepatitis C virus (HCV), supporting that HSPA6 may assist the function of cellular protein(s) generally required for viral IRES activities. This is the first report functionally demonstrated that HSPA6, a novel inducible HSP70 member, impacts a viral life cycle.

## Materials and Methods

### Cells and Viruses

RD (Human rhabdomyosarcoma; ATCC^®^, CCL-136^TM^), HEK293T, HeLa and Huh7 cells were cultured in Dulbecco’s modified Eagle’s medium (DMEM, Gibco) containing 100 unit/ml penicillin, 10 μg/ml streptomycin, 0.25 μg/ml amphotericin B, 2 mM L-glutamine and 10% fetal bovine serum (HyClone) at 37°C with 5% CO_2_. The EV-A71 genotype C strain (4643/Taiwan/1998), provided by Dr. Shin-Ru Shih (Chang-Gung University, Taiwan), was amplified in RD cells. All experiments using infectious viruses were carried out in biosafety level 2 laboratory, following the guidelines of the Center of Environmental Protection and Safety and Health (National Yang-Ming University, Taiwan).

### Plasmids

The detailed construction of R1 replicon has been described previously (Su et al., 2020). Briefly, for R1 3D^*D*330*A*^, in which Asp330 was mutated to an Ala to disrupt the enzymatic activity, was constructed by PCR-based site-directed mutagenesis using the R1 replicon as a template (Su et al., 2020). The monocistronic reporter plasmid (IRES-Luc), which contains EV-A71 IRES at the 5′end and drives the translation of the luciferase protein, was described previously (Su et al., 2018). To construct pFlag-CMV2-HSPA6, *HSPA6* gene was amplified from the cDNA of EV-A71 infected RD cells by nested PCR and then cloned into pFlag-CMV2 (Sigma-Aldrich) at *Not*I and *Kpn*I sites to generate pFlag-HSPA6. A wobble HSPA6 mutation (nucleotides 7–9 GCC→GCA), which resists the action of sgRNA used in knockout cells, was generated by site-direct mutagenesis. HCV IRES-Luc was previously described (Su et al., 2018). CV-A16 IRES-Luc and Echo 9 IRES-Luc were gifts from Dr. Szu-Hao Kung (National Yang-Ming University, Taiwan) (Hou et al., 2016). EMCV IRES-Luc was generated by inserting firefly *Luc* gene into the pTM1 plasmid (Addgene).

### Antibodies

Commercial antibodies used in this study were mouse anti-EV-A71 VP1 (Abnova), mouse anti-EV-A71 VP0/VP2 (Millipore), mouse anti-HSPA6 (Enzo), sheep anti-mouse IgG conjugated to horse radish peroxidase (HRP) (GE Healthcare) and goat anti-mouse IgG (H+L) conjugated to Alexa Fluor Plus 488 (Invitrogen). The mouse antisera against EV-A71 2C, 3C^*pro*^ and 3D^*pol*^ were home made using purified recombinant 6× His-tagged proteins from *Escherichia coli*.

### Western Blot Analysis

Cells were lysed in RIPA buffer (pH 7.4, 50 mM Tris–HCl, 150 nM NaCl, 1% NP-40, 0.1% SDS, 0.1% DOC, 2 mM EDTA, 10 mM NaF, 1 mM Na_3_VO_4_, 1 mM PMSF, 50 μM leupeptin, 50 μM aprotinin), mixed with cracking buffer (pH 6.8, 250 mM Tris–HCl, 40% glycerol, 20% β-ME, 8% SDS, 0.04% bromophenol blue), and resolved by 7.5 or 10% SDS-polyacrylamide gels. For viral protein detection, 50 μg of lysate was used, while for HSPA6 detection, 75 μg of lysate was used. Gels were then electro-transferred onto polyvinylidene difluoride membranes at 120 V for 3 h for viral proteins or 55 V for 16 h for HSPA6 (transfer buffer: 25 mM Tris, 192 mM glycine, 20% methanol). After blocked in TBS-T [50 mM Tris–HCl (pH 7.6), 150 mM NaCl, 0.1% Tween-20] containing 5% non-fat milk, the membranes were incubated with various primary antibodies. For HSPA6 detection, membrane was incubated with anti HSPA6 antibody (1:100) for 32 h, followed by incubation with secondary antibody for 16 h. Signals were detected with ECL Western blotting detection reagent.

### Quantitative RT-PCR (RT-qPCR) and *in vitro* Transcription

Total RNA was extracted using TRizol (Invitrogen), followed by reverse transcription using Hiscript I reverse transcriptase (Bionovas). 1/12 of the total cDNA was subjected to qPCR with primers specific to 3D^*pol*^ (vRNA), GAPDH (internal control), and luciferase reporter plasmids (Supplementary Table 1 for primer sequences) using Fast SYBR Green Master Mix (Applied Biosystems). For generating *in vitro* transcribed RNA, plasmids IRES-Luc (*Xba*I), R1 3D^*D*330*A*^ (*Sal*I), EMCV IRES-Luc (*Sal*I), and HCV IRES-Luc (*Sal*I), CV-A19 IRES-Luc (*Sca*I), and Echo 9 IRES-Luc (*Sca*I), linearized by restriction enzyme specified in the parentheses, were used as templates and transcribed using MEGAscript T7 transcription kit (Invitrogen). 2 μg of the RNA was transfected into cells using Lipofectamine 3,000 reagent (Invitrogen). Cells were harvested for RT-qPCR or luciferase assay at the time point specified in each figure.

### Lentivirus-Based HSPA6 Knockdown and Knockout

For HSPA6 knockdown, lentiviruses expressing HSPA6 shRNA (clone 1: GATGTGTCGGTTCTCTCCATT and clone2: GAGCAGTACAAGGCTGAGGAT) were used to infect RD cells. Puromycine (2.5 μg/ml) resistant RD cells were used for subsequent experiments. For *HSPA6* knockout, CRISPR/Cas9 system for *HSPA6* knockout (sgRNA: GCCCACCGCGAGCTCCCGTG) and its control were purchased from National RNAi Core Facility (Academia Sinica, Taiwan). After puromycin (2.5 μg/ml) selection, clones are amplified from single RD cell isolated by limiting dilution. To validate *HSPA6* KO, cells were challenged with heat shock condition (42°C, 2 h) and collected for Western analysis using anti HSPA6 antibody (Enzo).

### Virus Titration

The supernatant containing extracellular viruses was collected from the EV-A71 infected RD cell culture and centrifugated at 5,700 × g for 5 min to remove cell debris. Cell associated viruses were prepared from cell lysates collected after freeze-and-thaw cycles and centrifugation at 15,300 × *g* for 10 min. The infectious viral titer was measured using fifty-percent tissue culture infective dose (TCID_50_) according to the Reed-Muench method.

### Viral Entry Detection

RD cells cultured on poly-L-Lysine coated coverslips were pretreated with 100 μg/ml cycloheximide for 1 h to prevent virus translation before viral infection, and then challenge with EV-A71 (MOI = 300) for another 1 h to allow viral internalization in the presence of cycloheximide. In the JG40 control experiment, JG40 (5 μM) was added together with cycloheximide. The cells were washed three times with PBS and fixed with ice-cold methanol for 3 min. After another PBS wash, the cells were permeabilized with 0.2% Triton X-100 at room temperature for 20 min, and then incubated with blocking buffer (5% FBS and 0.75% BSA in PBS) at room temperature for 1 h. The cells were treated with mouse antibody against EV-A71 VP0/VP2 (Millipore) at room temperature for 2 h, and stained with goat anti-mouse IgG (H + L) conjugated to Alexa Fluor Plus 488 (Invitrogen) at room temperature for 2 h. Cell nuclei were stained with 0.5 nM DAPI, prior to mounting on glass slides. The specimens were observed by confocal microscopy (ZEISS LSM 700). The signal intensity was quantified using Metamorph software.

### Luciferase Reporter Assay

The detailed construction of IRES-Luc (Su et al., 2018) and R1 3D^*D*330*A*^ (Su et al., 2020) were previously described. 1 μg of *in vitro* transcribed reporter RNA of IRES-Luc or R1 3D^*D*330*A*^ was transfected into *HSPA6* KO and knockdown RD cells. HSPA6 shRNA #2 was used for knocking down HSPA6 in the RD cells used in the luciferase assays. The cell lysates were harvested at time points indicated. 2 μg of *in vitro* transcribed reporter RNA of the CV-A19 IRES-Luc, Echo 9 IRES-Luc and EMCV IRES-Luc were transfected into HeLa cells; and the reporter RNA of HCV IRES-Luc was transfected into Huh7 cells. The cell lysates were harvested at 6 h post transfection. One third of cell lysates were used for luciferase activity using Bright-Glo^TM^ Luciferase assay system (Promega). One half of the cell lysates were used for RNA purification, which was subjected to RT-qPCR and then used for the normalization of transfection efficiency. Luciferase activity was normalized with RNA transfected to reflect IRES activity.

### Cycloheximide Chase

To monitor the stability of viral proteins, the RD cells were treated with 100 μg/ml cycloheximide (CHX) at 9 h post EV-A71 infection, and then the cell lysates were harvested at 1, 2, 4, and 6 h post cycloheximide treatment. The equal volume of each sample was loaded into SDS-PAGE for Western blot analysis using antisera against non-structural proteins 2C, 3C^*pro*^, and 3D^*pol*^. The signal intensity of each band was quantified by ImageJ software and plotted as the value taken 1 h CHX treatment as 100%. The linear regression curves were charted by GraphPad Prism five software.

### Statistics Analysis

All the data analyses and statistical graphs were processed by GraphPad Prism five software. The statistical methods used in this study were unpaired Student’s *t*-test or one-way ANOVA followed by Dunnett’s *post hoc* test. A *P*-value < 0.05 is regarded as statistically significant. (^∗∗∗^*P* < 0.001, ^∗∗^*P* < 0.01, and ^∗^*P* < 0.05).

## Results

### HSPA6 Is Induced to Support EV-A71 Replication Cycle

**FIGURE 1:**
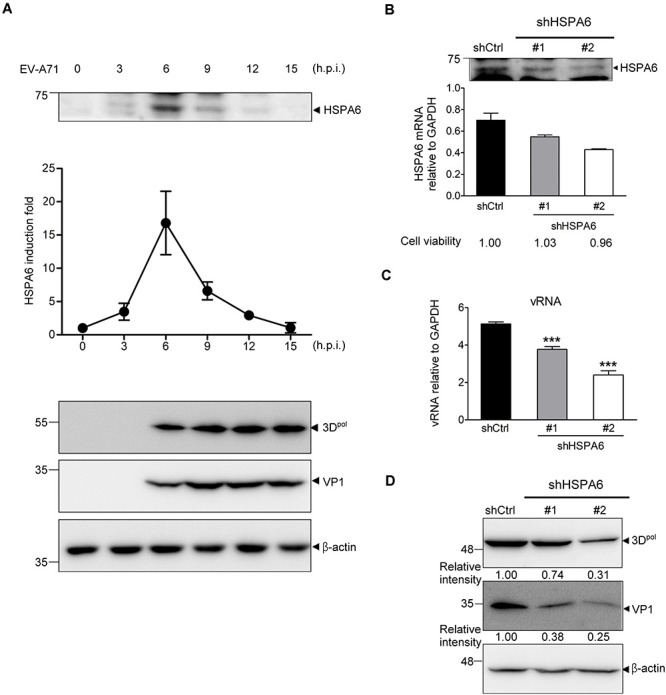
HSPA6 is induced to support EV-A71 replication cycle. **(A)** RD cells were infected with EV-A71 (MOI = 5) and harvested for the protein expression of HSPA6 and viral 3D^*pol*^ at the time points indicated. HSPA6 protein expression was quantitated from three independent experiments and presented as induction fold over 0 time point. **(B–D)** RD cells were infected with lentivirus expressing control shRNA (shCtrl) or shRNA specific to HSPA6 (shHSPA6 clone #1 and clone #2). 48 h later, the cells were infected with EV-A71 (MOI = 5) and harvested at 6 hpi for analyzing the productions of **(B)** HSPA6 mRNA, **(C)** cell-associated vRNA, and **(D)** viral proteins 3D^*pol*^ and VP1. Cell viability of shHSPA6-treated cells was also indicated in **(B)**. HSPA6 mRNA and vRNA were measured by RT-qPCR and normalized with GAPDH mRNA. Protein production was examined by Western blot analysis using specific antibody indicated. RT-qPCR results were presented as means ± standard deviation (SD) (*n* = 3). One-way ANOVA followed by Dunnett’s *post hoc* analysis was performed to compare the differences between the shCtrl group and the shHSPA6 groups in **(C)**. ***P < 0.001.

In the previous study, we have reported that HSP70 family proteins, including HSPA1, HSPA8 and HSPA9, played positive roles in EV-A71 life cycle (Su et al., 2020). Here we report that an additional HSP70 member, HSPA6, was induced and reached to a peak at 6 h post infection (hpi) (Figure 1A and Supplementary Figure 1). The HSPA6 protein induction from three biological repeats was quantified (Figure 1A). To understand the importance of this induction, we infected RD cells with a lentivirus carrying the shRNA specific to HSPA6, followed by EV-A7 infection 48 h post lentivirus transduction. While both shRNA clones (#1 and #2) reduced amount of HSPA6 mRNA and protein, shRNA #2 gave a better knockdown efficiency in RD cells (Figure 1B). Both viral RNA (vRNA) levels (Figure 1C) and viral protein production of 3D^*pol*^ and VP1 (Figure 1D) were decreased in HSPA6-depleted cells. HSPA6 depletion did not reduce cell viability (Figure 1B). Notably the reduction was proportional to the knockdown efficiency, suggesting a positive role of HSPA6 in EV-A71 life cycle.

### HSPA6 Is a Positive Regulator for EV-A71 Life Cycle

**FIGURE 2:**
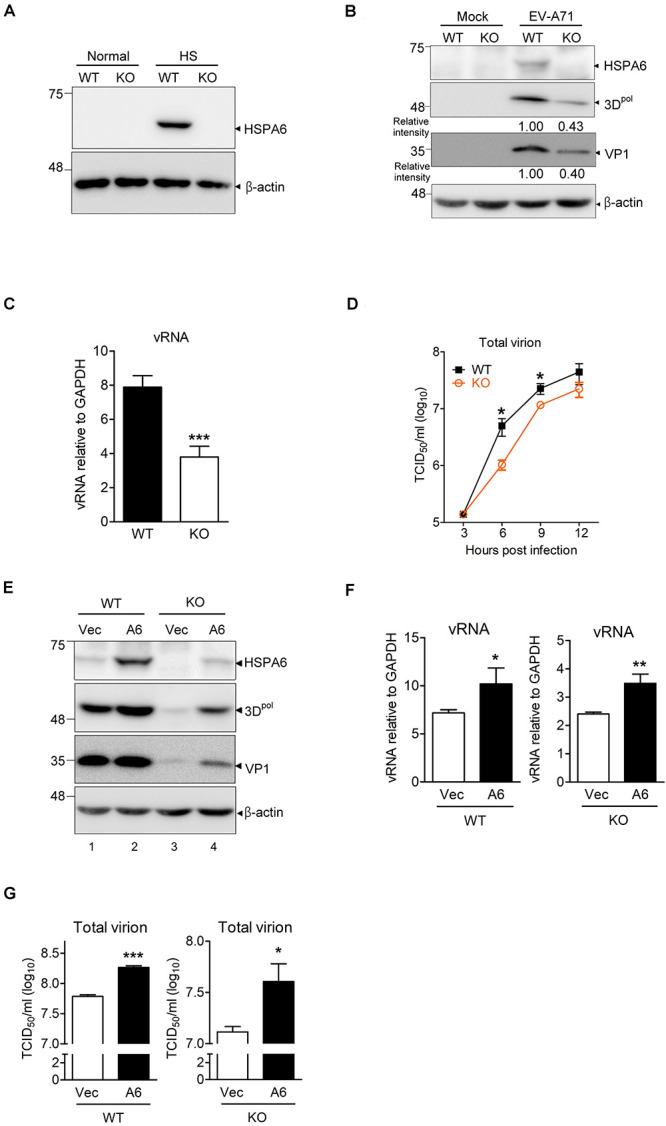
HSPA6 is a positive regulator for EV-A71 cycle. *HSPA6* KO RD cells were generated using the CRISPR/Cas9 approach. **(A)** HSPA6 protein production was examined in WT RD and *HSPA6* KO RD cells cultured in normal condition (37°C) or heat shock condition (42°C) for 2 h. **(B–D)** The WT RD and *HSPA6* KO RD cells were infected with EV-A71 (MOI = 5). Cell lysates were prepared for the analyses of **(B)** HSPA6 and viral proteins 3D^*pol*^ and VP1, and **(C)** cell-associated vRNA at 6 hpi. **(D)** Total virion were prepared from culture supernatant and cell lysates collected at 3, 6, 9, and 12 hpi and then titrated. **(E–G)** WT RD and *HSPA6* KO RD cells were transfected with vector control (Vec) or Flag-HSPA6 expressing plasmid (A6) (0.2 μg/6 × 10^5^ cells). 24 h post transfection, cells were infected with EV-A71 (MOI = 5) and harvested for the analyses of **(E)** viral proteins 3D^*pol*^ and VP1, **(F)** cell-associated vRNA at 6 hpi, and **(G)** total viral titer at 12 hpi. Protein production was measured by Western analysis using specific antibody indicated. HSPA6 mRNA and vRNA were measured by RT-qPCR and normalized with GAPDH mRNA. Viral titer was determined by a 50% tissue culture infective dose (TCID_50_) assay. Data were presented as means ± SD (*n* = 3) and compared with unpaired Student’s *t*-test. ***P < 0.001, **P < 0.01, and *P < 0.05.

Because using shRNA only achieved partial depletion of HSPA6 in RD cells (Figure 1B), we therefore generated an *HSPA6* knockout (KO) RD clone using the CRISPR/Cas9 approach. HSPA6 protein production was examined among cell lysates prepared from wild-type (WT) and *HSPA6* KO RD cells with or without heat treatment. While HSPA6 protein was highly induced in WT RD cells under the heat shock (HS) condition, it became undetectable in *HSPA6* KO cells (Figure 2A), indicating HSPA6 was well depleted. To better understand how HSPA6 impacts EV-A71 life cycle, the levels of viral protein, vRNA and viral titer were compared between WT and *HSPA6* KO RD cells infected with EV-A71. The results showed that 3D^*pol*^ and VP1 proteins were produced at ∼40% in *HSPA6* KO cells as compared to those in WT cells (Figure 2B). Cell-associated viral RNA was reduced to 50% in *HSPA6* KO cells as well (Figure 2C). Total virions, which included cell-associated and extracellular viral particles, were collected from 3 to 12 hpi at 3-h intervals and then titrated. The data revealed that viral production was reduced in the absence of HSPA6 protein at all-time points and with a biggest difference (∼4.8-fold) at 6 hpi (Figure 2D). Put together, these data indicate that HSPA6 has a positive role in EV-A71 life cycle.

To rule out that the reductions in viral protein, vRNA and titer were due to off-target effects of the sgRNA, we conducted rescue experiments. We transfected *HSPA6* KO RD cells with a small amount of HSPA6-expressing plasmid (0.2 μg/6 × 10^5^ cells) to restore HSPA6 expression close to its endogenous levels before EV-A71 infection (Figure 2E, compare lanes 1 and 4). The cells were harvested at 6 hpi for the analysis of viral proteins (3D^*pol*^ and VP1) and cell associated vRNA, and at 12 hpi for the measurement of total viral titers. The results indicated that ectopic HSPA6 expression in WT RD further increased 3D^*pol*^ level (Figure 2E), vRNA (Figure 2F), and viral titer (Figure 2G). More importantly, it also restored these three infection indices in *HSPA6* KO RD cells (Figures 2E–G). These data demonstrated that the reduction of viral propagation in *HSPA6* KO RD cells was not caused by off-target effects. Thus, we concluded that HSPA6 facilitates EV-A71 life cycle.

### HSPA6 Is Not Needed for EV-A71 Internalization

**FIGURE 3:**
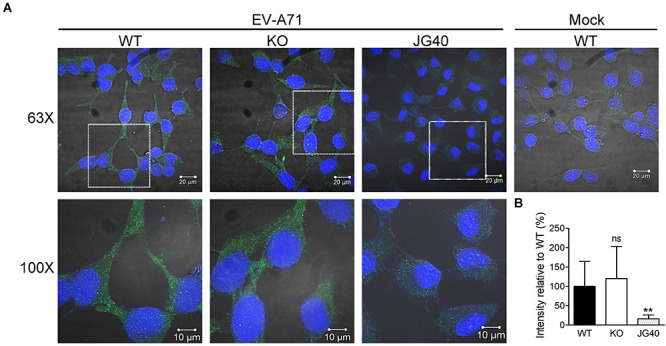
HSPA6 is not needed for EV-A71 internalization. WT RD or *HSPA6* KO RD cells, pretreated with cycloheximide (CHX, 100 μg/ml) to prevent viral RNA translation, were infected with EV-A71 (MOI = 300). JG40 (5 μM), an EV-A71 inhibitor, was used as a control. One hour later, the cells were fixed, permeabilized, and incubated with anti-VP0/VP2 primary antibody, followed by Alexa Fluor 488 labeled secondary antibody. **(A)** Cell images captured by confocal fluorescent microscopy at 63× and 100× were shown (green: VP0/VP1; blue: nuclei). **(B)** The integrated green fluorescence intensities, averaged from 10–15 fields (20–30 cells/field), were compared between signals from WT RD and *HSPA6* KO RD cells. The results, presented as means ± SD of one set of images, are shown here. Similar results were obtained from at least two other sets of experiments. Unpaired Student’s *t*-test was performed to compare the differences between WT and *HSPA6* KO cells. ns, not significant.

We next investigated at which stage of the EV-A71 life cycle HSPA6 was involved. RD cells were pretreated with cycloheximide (CHX) for 1 h before EV-A71 infection. To study if HSPA6 plays a role in EV-A71 internalization, the CHX treated cells were then infected with EV-A71 for 1 h to allow viral particles internalization and then harvested immediately for immunofluorescence staining using antibody against EV-A71 VP0/VP2. The CHX pre-treatment prevented vRNA translation upon viral entry, therefore any VP0/VP2 signals detected would be from the entering viral particles exclusively. JG40, an EV-A71 inhibitor (Su et al., 2020), was used as a control. The images of confocal microscopy (Figure 3A) as well as their quantification data (Figure 3B) showed no significant difference in VP0/VP2 signals between WT and *HSPA6* KO cells, suggesting that HSPA6 is not needed for EV-A71 entry into RD cells.

### HSPA6 Is a Positive Regulator for EV-A71 IRES-Mediated Translation

**FIGURE 4:**
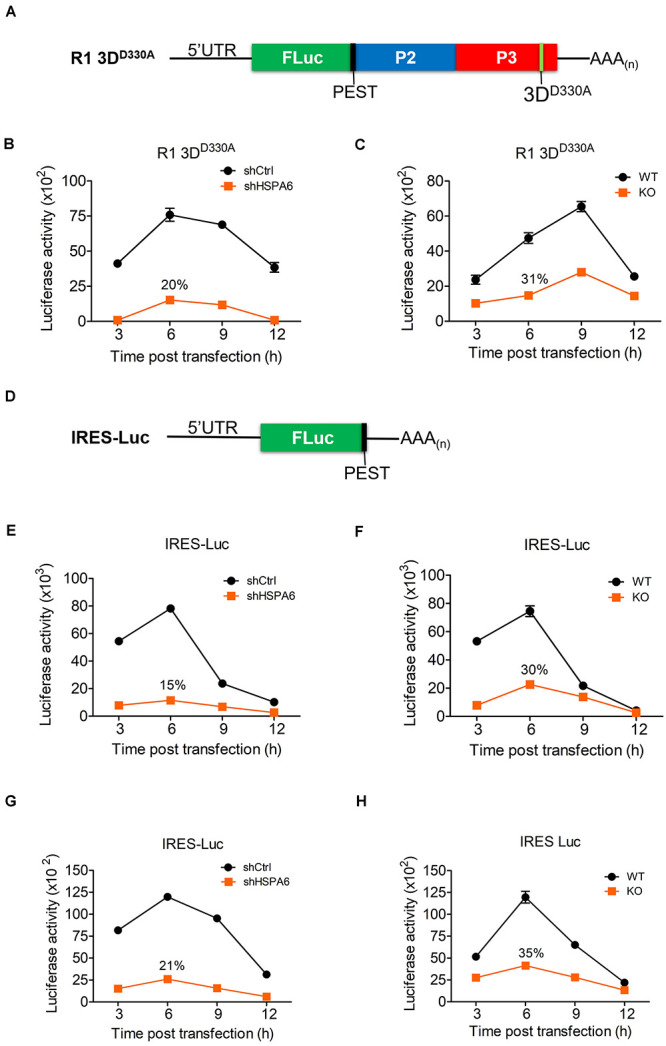
HSPA6 upregulates EV-A71 IRES-meditated translation. **(A)** Schematic representation shows the mutant replicon R1 3D^*D*330*A*^. This replicon has a D330A mutation in the 3D^*pol*^ region a PEST (Pro, Glu, Ser, and Thr) sequence, which caused rapid degradation of the luciferase protein. The *in vitro* transcribed RNA of R1 3D^*D*330*A*^ was transfected into knockdown control (shCtrl) RD, shHSP6A RD, WT RD, and HSPA6 KO RD cells. Cells were harvested at 3, 6, 9, and 12 h post transfection for luciferase assay. Luciferase activity of reporter R1 3D^*D*330*A*^ was compared between **(B)** shCtrl RD and shHSPA6 RD cells and between **(C)** WT RD and *HSPA6* KO RD cells. **(D)** Schematic representation shows the reporter construct IRES-Luc, which contains no additional EV-A71 genes. **(E,F)** The *in vitro* transcribed RNA of IRES-Luc was transfected into RD cells 6 h post EV-A71 infection (MOI = 5). Cells were harvested at 3, 6, 9, and 12 h post transfection for luciferase assay. Luciferase activity was compared between **(E)** shCtrl RD and shHSP6A RD cells, and **(F)** WT RD and *HSPA6* KO RD cells. **(G,H)** The *in vitro* transcribed RNA of IRES-Luc was transfected into RD cells without EV-A71 infection. Cells were harvested at 3, 6, 9, and 12 h post transfection for the luciferase activity assay. Luciferase activity was compared between **(G)** shCtrl RD RD and shHSPA6 RD cells and between **(H)** WT RD and *HSPA6* KO RD cells. All measurements were normalized with RNA transfection efficiency. The data were presented as means ± SD (*n* = 3) and analyzed with unpaired Student’s *t*-test. ***P < 0.001, **P < 0.01, and *P < 0.05.

The immediate step following its entry and uncoating is the translation of vRNA mediated by the IRES during EV-A71 life cycle. We first examined if RNA transfection had any impact on HSPA6 protein expression and found that HSPA6 was induced (Supplementary Figure 2). To examine the potential roles of HSPA6 in EV-A71 IRES-mediated translation, an R1 3D^*D*330*A*^ mutant replicon (Figure 4A) was firstly used. This replicon contains a luciferase reporter replacing the P1 region of the EV-A71 genome and has a D330A mutation in the 3D^*pol*^ region. A PEST (Pro, Glu, Ser, and Thr) sequence was added at the carboxyl terminus of luciferase, which caused rapid degradation of the luciferase protein and therefore minimized protein accumulation. This allows the Luc activity measured to reflect the translational activity at the moment. In addition, the enzymatic mutation of D330A in the 3D^*pol*^ region prevents amplification of the replicon (Jiang et al., 2011) and the lack of P1 structure protein region avoids the formation of new viral particles, thus the luciferase activity would solely reflect translational activity. The *in vitro* transcribed RNA of R1 3D^*D*330*A*^ mutant replicon was transfected into knockdown control (shCtrl) and HSPA6 knockdown RD cells (Figure 4B), or WT and *HSPA6* KO RD cells (Figure 4C). The luciferase activity was measured at 3, 6, 9, and 12 h post transfection and normalized with the RNA levels transfected in each cell line. The luciferase activity was significantly lower in both HSPA6 knockdown (Figure 4B) and *HSPA6* KO cells (Figure 4C) at each time point as compared to that in control cells. The luciferase activity was reduced to 20 and 31% at 6 h post transfection in HSPA6 knockdown and *HSPA6* KO cells, respectively. Since HSPA6 was induced during EV-A71 infection, we first examined the function of HSPA6 in IRES activity during EV-A71 infection. The *in vitro* transcribed RNA of IRES-Luc, which is a Luc reporter containing no additional viral genes (Figure 4D), was transfected at 6 hpi into EV-A71-infected knockdown control (shCtrl) and HSPA6 knockdown RD cells (Figure 4E), or WT and *HSPA6* KO RD cells (Figure 4F). Cells were harvested for luciferase activity measurement at 3, 6, 9, and 12 h post transfection. Similarly, the luciferase activity was significantly lower in both HSPA6 knockdown (Figure 4E) and *HSPA6* KO cells (Figure 4F) at each time point as compared to that in control cells. The luciferase activity was reduced to 15 and 30% at 6 h post transfection in HSPA6 knockdown and *HSPA6* KO cells, respectively. These results demonstrated that HSPA6 played a positive role in EV-A71 IRES activity during infection. It is known that IRES-mediated translation of EV-A71 could be regulated by either cellular ITAFs or viral proteins (Dong et al., 2018), whose folding is governed by HSP70s (Su et al., 2020). Thus, we asked whether HSPA6 could stimulate IRES activity without the presence of viral proteins. The *in vitro* transcribed RNA of IRES-Luc was transfected into cells without EV-A71 infection and luciferase activity was measured at 3, 6, 9, and 12 h post transfection. The results showed that in the absence of EV-A71 viral proteins, comparable patterns of reduction in IRES-mediated luciferase activity were found in both HSPA6 knockdown (Figure 4G) and *HSPA6* KO cells (Figure 4H) as those in the presence of EV-A71 infection. The luciferase activity was reduced to 21 and 35% in HSPA6 knockdown and *HSPA6* KO cells at 6 h post transfection, respectively, suggesting that HSPA6 possibly modulates IRES activity through regulating cellular factors rather than viral proteins.

### HSPA6 Depletion Does Not Influence EV-A71 Replication Efficiency

**FIGURE 5:**
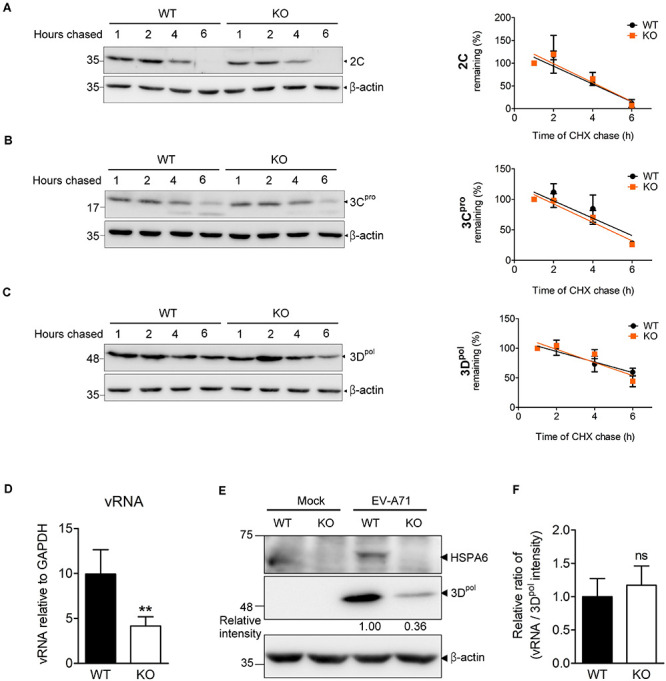
HSPA6 depletion does not affect the protein stabilities of viral 2C, 3C^*pro*^, and 3D^*pol*^ proteins, nor does it reduce EV-A71 replication efficiency. WT RD and *HSPA6* KO RD cells were infected with EV-A71 (MOI = 5) and then treated with CHX (100 μg/ml) at 9 hpi to stop translation. The remaining viral proteins **(A)** 2C, **(B)** 3C^*pro*^, and **(C)** 3D^*pol*^ at 1, 2, 4, and 6 h after CHX treatment were examined by Western blot analysis (left panels). At each time point, the intensity for band specific to each protein from the Western analysis was quantified by ImageJ software. The value of each band was normalized with β-actin and plotted for the slope of linear regression curve. The band intensity from 1 h post CHX chase was arbitrarily set as 100% (right panels). **(D–F)** WT RD and *HSPA6* KO RD cells were infected with EV-A71 (MOI = 5) and harvested for the analyses of **(D)** cell-associated vRNA and **(E)** viral protein 3D^*pol*^ at 6 hpi. **(F)** A normalization of cell-associated vRNA with viral polymerase 3D^*pol*^ expression (vRNA/3D^*pol*^) was calculated to represent the replication efficiency in WT RD and *HSPA6* KO RD cells. The data were presented as means ± SD (*n* = 3) and analyzed by unpaired Student’s *t*-test. ns, not significant.

During the infection cycle of positive sense RNA viruses including EV-A71, it is technically difficult to separate the replication period from the translation period because these two steps are tightly linked and overlapped. Depletion of HSPA6 reduced IRES-mediated translation (Figure 4), which would lead to the reduction of viral RNA synthesis naturally due to the decrease in viral protein production. As a consequence, we were unable to determine if HSPA6 has a role in genome replication by simply measuring the vRNA level. However, as a protein chaperone, HSPA6 may affect viral replication through helping proper folding of the viral proteins constituting replication complex. Thus, we investigated whether HSPA6 stabilized replication complex components 2C, 3C^*pro*^, and 3D^*pol*^ by CHX chase assay. EV-A71-infected WT RD and *HSPA6* KO RD cells were treated with translation inhibitor CHX at 9 hpi. The remaining 2C, 3C^*pro*^, and 3D^*pol*^ were chased for another 1, 2, 4, and 6 h and measured by Western analysis (Figures 5A–C and Supplementary Figure 3); in addition, the mean intensity of three sets of experiments for bands specific for 2C, 3C^*pro*^, and 3D^*pol*^ was plotted. There were no significant differences in protein stability between 2C (Figure 5A), 3C^*pro*^ (Figure 5B), and 3D^*pol*^ (Figure 5C) prepared from WT RD and *HSPA6* KO RD cells. Consistently, a normalization of cell-associated vRNA levels (Figure 5D) with viral 3D^*pol*^ expression (Figure 5E) at 6 hpi, deduced as replication efficiency, showed no differences between WT RD and *HSPA6* KO RD cells (Figure 5F). Collectively, these data suggest that HSPA6 does not regulate EV-A71 replication.

### Knockout of HSPA6 Does Not Reduce Virion Assembly and Release Efficiency of EV-A71

**FIGURE 6:**
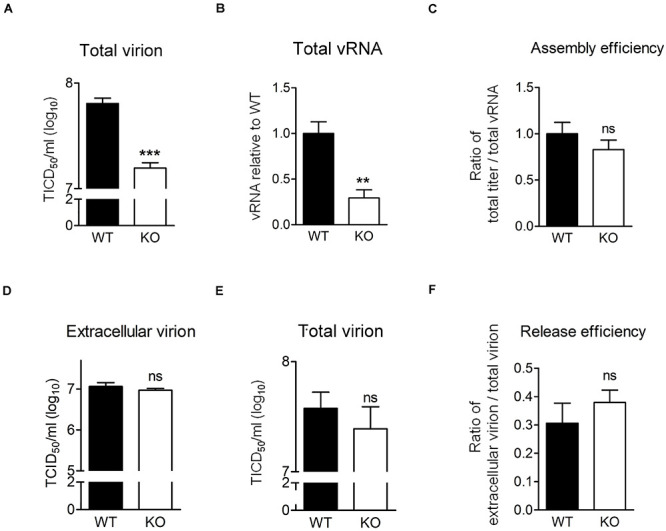
HSPA6 depletion does not reduce virion assembly and release efficiency of EV-A71. **(A–C)** WT RD or *HSPA6* KO RD cells were infected with EV-A71 (MOI = 5). At 9 hpi, **(A)** total virion and **(B)** total vRNA were prepared from the culture supernatant together with cell lysates for TCID_50_ assay and RT-qPCR, respectively. **(C)** The assembly efficiencies, represented by the ratio of titer of total virion over total vRNA, are shown. **(D–F)** WT RD or *HSPA6* KO RD cells were infected with EV-A71 (MOI = 5). At 12 hpi, **(D)** the extracellular virion from supernatant and **(E)** total virion were harvested and titrated using TCID_50_ assay. **(F)** The release efficiencies, represented by the ratio of extracellular virion over total virion, are shown. The results were presented as means ± SD (*n* = 3) and analyzed by unpaired Student’s *t*-test. ***P < 0.001, **P < 0.01. ns, not significant.

We showed that EV-A71 production was lower in the absence of HSPA6 (Figure 2D) and higher in the presence of additional HSPA6 (Figure 2G). It is possible that other than its roles in IRES-mediated translation, HSPA6 is involved in viral assembly or release. To explore whether HSPA6 affects EV-A71 particles formation, WT RD and *HSPA6* KO RD cells were challenged with EV-A71, and the total virions and total viral RNA prepared from cell-associated cells and extracellular viral particles were examined at 9 hpi. As expected, the total production of virions (Figure 6A) and vRNA (Figure 6B) were significantly reduced in *HSPA6* KO RD cells since HSPA6 affects viral translation. To separate the effects of HSPA6 knockout on viral assembly from IRES-mediated translation, the ratio of total viral particle over total vRNA (virion/vRNA) was used to represent assembly efficiency. The result showed that the assembly efficiency of EV-A71 is similar in WT RD and *HSPA6* KO RD cells (Figure 6C), suggesting that HSPA6 does not regulate EV-A71 virion assembly. Finally, we asked whether HSPA6 was involved in EV-A71 virion release. We considered the ratio of extracellular virion to total virion as release efficiency. The extracellular virion from culture supernatant and total virion at 12 hpi were harvested from WT RD and *HSPA6* KO RD cells infected with EV-A71. There were no differences found in viral titers of extracellular virion (Figure 6D), total virion (Figure 6E), and release efficiency (Figure 6F) at 12 hpi between WT RD and *HSPA6* KO RD cells. Put together, we concluded that HSPA6 played no role in either EV-A71 assembly and release.

Finally, we asked whether HSPA6 was involved in EV-A71 virion release. We considered the ratio of extracellular virion to total virion as release efficiency. The extracellular virion from culture supernatant and total virion were harvested at 12 hpi from WT RD and *HSPA6* KO RD cells infected with EV-A71. There were no significant differences found in viral titers of extracellular virion (Figure 6D), total virion (Figure 6E), and release efficiency (Figure 6F) between WT RD and *HSPA6* KO RD cells. Put together, we conclude that HSPA6 plays no role in either EV-A71 assembly or release.

### HSPA6 Facilitates IRES Activities From Other Viruses

**FIGURE 7:**
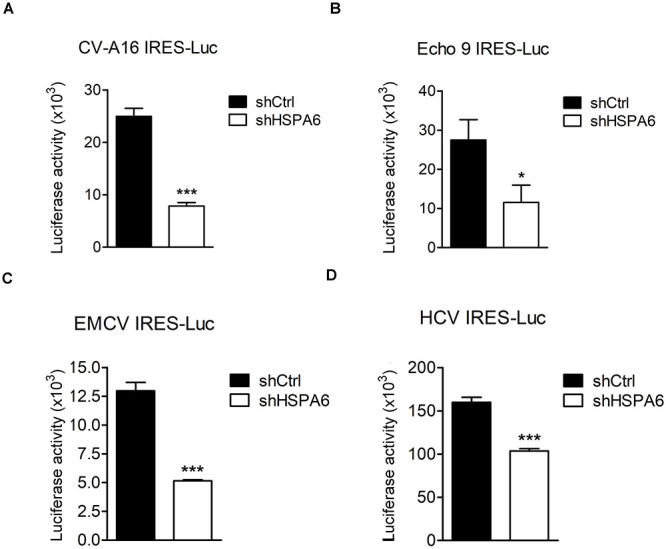
HSPA6 facilitates IRES activities from other viruses. HeLa and Huh7 cells were first infected with shCtrl or shHSPA6 expressing lentiviruses, followed by puromycin selection for 48 h to generate HSPA6-knockdown cells. The *in vitro* transcribed RNA of **(A)** CV-A16 IRES-Luc, **(B)** Echo 9 IRES-Luc, **(C)** EMCV IRES-Luc, or **(D)** HCV IRES-Luc was transfected into HeLa cells, whereas the RNA of HCV IRES-Luc was transfected into Huh7 cells. Luciferase activities expressed in WT RD cells and HSPA6-knockdown cells were compared at 6 h post transfection. All the values of luciferase activity were normalized with the RNA transfection efficiency. The data were presented as means ± SD (*n* = 3), and analyzed with unpaired student’s *t*-test. ***P < 0.001, and *P < 0.05.

The IRES-Luc activity was reduced comparably in HSPA6-depleted cells in the presence (Figures 4E,F) or absence (Figures 4G,H) of EV-A71 infection, suggesting that HSPA6 facilitated the IRES activity of EV-A71 through chaperoning cellular proteins rather than viral proteins. We then asked if HSPA6 could influence the IRES activities of other viruses. To address this question, individual luciferase reporter driven by the IRES from CV-A16, echovirus 9, EMCV, or HCV was used. The *in vitro* transcribed reporter RNA was transfected into HSPA6-knockdown HeLa cells except the RNA of HCV IRES-Luc that was transfected into HSPA6-knockdown Huh7 cells. The luciferase activities were examined at 6 h post transfection and normalized with amounts of RNA transfected. Intriguingly, the results showed that the luciferase activity was reduced for all four IRES-Luc constructs in HSPA6-knockdown cells (Figures 7A–D), suggesting that HSPA6 may assist the function of cellular proteins commonly required for these viral IRES activities.

## Discussion

Viral proteins are made in a large quantity in a short period of time during viral infection. To ensure proper folding of viral proteins, viruses often manipulate cellular chaperone proteins in favor of their own replication. The HSP70s, composed of a large chaperone protein family, play important roles in maintaining cellular homeostasis (Saibil, 2013). On the other hand, their participation in viral replication cycle is often a result of viral exploitation (Morimoto et al., 1997; Santoro et al., 2010; Wan et al., 2020). In our previous study, we found that HSPA1, HSPA8 and HSPA9, three major members of HSP70, play multiple functions in the EV-A71 life cycle (Su et al., 2020). Here we further reported that HSPA6, a scarcely studied HSP70 family member, was also involved in EV-A71 infection. Being a strictly stress-inducible HSP (Noonan et al., 2007a, b), we found that HSPA6 protein production was induced by EV-A71 infection fittingly and reached a peak at the middle stage (6–9 hpi) of the infection cycle (Figure 1A). The knockout of HSPA6 led to the negative impacts on EV-A71 viral proteins, viral RNA and virions (Figures 2B–D), while the addition of HSPA6 had the positive effects (Figures 2E–G), indicating that HSPA6 was a positive regulator for the EV-A71 life cycle. Unlike other HSP70 isoforms we reported, which usually participate in multiple stages of the EV-A71 life (Su et al., 2020), HSPA6 was required for the IRES-mediated translation only (Figures 3–6). Surprisingly, HSPA6 regulated the IRES activity in the absence of viral proteins (Figures 4G,H), suggesting that HSPA6 facilitated EV-A71 IRES activity through cellular proteins instead of viral proteins.

An interesting question raised here is how HSPA6 is induced. Viruses could regulate host HSPs at different levels such as transcription, translation, post-translational modification and cellular localization (Bolhassani and Agi, 2019). Our preliminary data indicated that the HSPA6 mRNA levels were greatly induced after EV-A71 infection (data not shown), suggesting that EV-A71 upregulated HSPA6 at least at the transcription level. The transcription of stress-inducible HSP70s can be controlled by heat shock factors (HSFs). A sequence analysis of HSP6A (HSP70B’) genome has found potential heat-shock elements (HSEs) recognized by HSFs in the promoter region starting at nucleotide residue −72 (transcription initiation site: +1). Additional HSEs located between −647 and +48 were identified in a subsequent study Under unstressed conditions, HSF is maintained as an inert monomeric form through its interactions with chaperones such as HSP90 and HSP70. In response to physiological stresses, HSF is released from chaperone and binds to HSEs in the HSP gene promoters (Morimoto, 1993, 1998). The nuclear localization activity of HSF can be further regulated by the phosphorylation mode carried out by calcium- or phospholipid-dependent protein kinase C (PKC) or by calcium/calmodulin-dependent protein kinase II (CaMKII) (Silver and Noble, 2012). Since EV-A71 infection induces calcium influx (Lu et al., 2013; Supasorn et al., 2020), we postulate that the calcium related protein kinases are activated upon viral infection to phosphorylate HSFs, leading to their nuclear entry. The binding of activated HSFs to the HSPA6 promoter may account for HSPA6 induction since multiple HSEs are present in its promoter region (Leung et al., 1990; Ramirez et al., 2015) (This paragraph has been shortened as requested by editor).

IRES-mediated translation is used by many viruses to avoid the shut off of cap-dependent translation caused by viral infection. Different types of IRES use distinct ITAFs for their individual IRES activities, but may share some common host factors (Hanson et al., 2012; Lee et al., 2017). For example, La protein (also known as Sjogren syndrome antigen B) has been found to regulate type I IRES activities from CV-B3 (Ray and Das, 2002), poliovirus (Meerovitch et al., 1989), and hepatitis A virus (cordes et al., 2008); it also regulates type II IRES from EMCV (Kim and Jang, 1999) and type III IRES from HCV (Costa-Mattioli et al., 2004). In this study, we found that HSPA6 was a positive regulator for EV-A71 IRES (type I)-mediated translation independent of viral proteins (Figure 4), suggesting that HSPA6 acts on cellular factors rather than viral proteins to modulate EV-A71 translation. Moreover, we found that two other type I IRESs from CV-A16 and echovirus 9 (Figures 7A,B), type II IRES from EMCV (Figure 7C), and type III IRES from HCV (Figure 7D) were also regulated by HSPA6, further suggesting that HSPA6 may act on a common ITAF or ITAFs. It has been reported that HSPA1 induces La protein production which in turn enhances the IRES-mediated translation of CV-B3 (Wang et al., 2017). Whether HSPA6 affects La protein production/function is unclear. Other examples of ITAFs including PTBP1, PCBP2 and hnRNP D (also known as AUF1) are known to regulate various types of viral IRESs as well [reviewed by Lee et al. (2017)] these ITAFs could therefore be the targets of HSPA6. Alternatively, yet-to-be-identified ITAFs regulated by HSPA6 could be responsible for the enhancement of the IRES activities. Another interesting question is how HSPA6 activates the IRES activity. It has been proposed that HSP70 chaperones enhance IRES activity by various mechanisms. To name a few possibilities, HSPA6 may facilitate the production, the folding and the transport of ITAFs, as well as the final assembly of translation-competent ribonucleoprotein complex. Alternatively, HSPA6 might enhance viral IRESs by its potential RNA binding activity since its association with HCV 3-NT′R was reported (Harris et al., 2006).

Both HSPA1 and HSPA6 are stress-induced HSP70s with high protein sequence homology (Daugaard et al., 2007; Noonan et al., 2007a). While HSPA6 may share overlapping functions with HSPA1, we showed that they have non-redundant function during EV-A71 infection. This is demonstrated by the knockout of *HSPA6* still reduced EV-A71 replication *via* reducing the IRES activity (Figures 2, 4). Consistently, our prior studies also showed that knockdown of HSPA1, in the presence of HSPA6, impaired several stages of EV-A71 life cycle (Su et al., 2020). In summary, we demonstrated not only that HSPA6 was induced during EV-A71 infection to facilitate its IRES-mediated translation, but also that it played roles in the IRESs from other viruses. There are limited studies about HSPA6, among them only one report showing its association with the 3′UTR of the HCV genome (Harris et al., 2006). We provide the very first report functionally, which demonstrates HSPA6, a novel inducible HSP70 member, impacts a viral life cycle.

## Data Availability Statement

The original contributions presented in the study are included in the article/Supplementary Material, further inquiries can be directed to the corresponding author/s.

## Author Contributions

Y-SS, L-HH, and C-JC conceived the project and wrote the manuscript. Y-SS conducted the experiments. All authors contributed to the article and approved the submitted version.

## Conflict of Interest

The authors declare that the research was conducted in the absence of any commercial or financial relationships that could be construed as a potential conflict of interest.
