# Supplementary material for: Heat Shock Protein A6, a Novel HSP70, Is Induced During Enterovirus A71 Infection to Facilitate Internal Ribosomal Entry Site-Mediated Translation
Source: Front Microbiol. 2021 May 7;12:664955. doi: 10.3389/fmicb.2021.664955 (PMC8137988; doi:10.3389/fmicb.2021.664955)
Supplement: Supplementary file 1 [file Data_Sheet_1.docx]

**Supplementary materials**

**Supplementary table S1. Primers used in this study**

| **Target gene** | **Sequences** |
| --- | --- |
| F-EV71/4643-3D | 5’-CCAAGATGAGCATGGAGGAT 3’ |
| R-EV71/4643-3D | 5’GATCTTGTCGATGGCCCTAA 3’ |
| F-Luc | 5’ CGTTATTTATCGGAGTTGCAGTTG 3’ |
| R-Luc | 5’ AAATCCCTGGTAATCCGTTTTAGA 3’ |
| F-GAPDH | 5’ GTATTGGGCGCCTGGTCACC 3’ |
| R-GAPDH | 5’ CGCTCCTGGAAGATGGTGATGG 3’ |
| F-HSPA6 | 5’GAGGAGGTGGAGAGGATGGTTCA 3’ |
| R-HSPA6 | 5’GCCTGTCCTCTTCGGGAATCTTG 3’ |

**Supplementary figures**

**
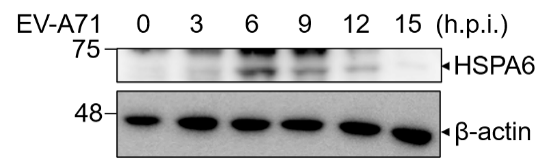
**

**
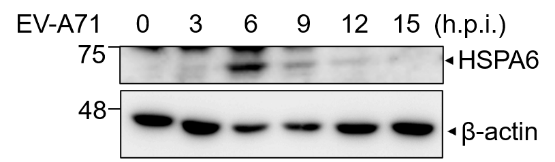
**

**Supplementary Figure S1. Biological replica for Figure 1A.** Two additional sets of the Western analyses showing that HSPA6 protein is induced upon EV-A71 infection.

**
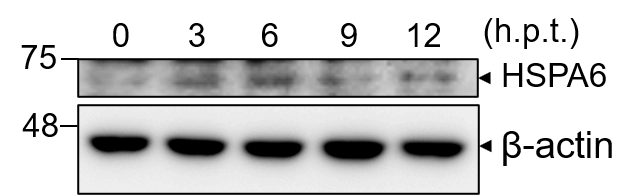
**

**Supplementary Figure S2. RNA transfection induced HSPA6 protein expression in RD cells.** Wild-type RD cells were transfected with IRES-Luc. Cells were harvested at 0, 3, 6, 9, and 12 h post transfection for Western analysis using anti-HSPA6 antibody.

**
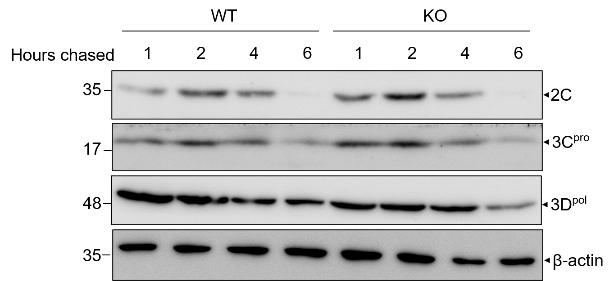
**

**
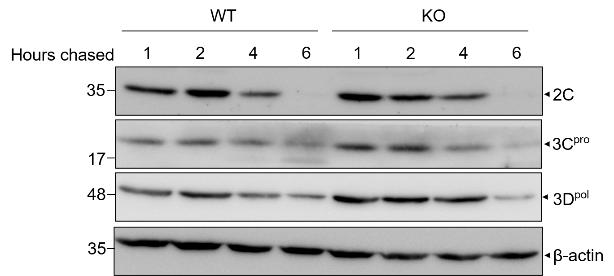
**

**Supplementary Figure S3. Biological replica for Figures 5A, 5B, and 5C.** Two additional sets of the Western analyses showing that knockout of HSPA6 protein did not affect the protein stability of viral proteins 2C,3C^pro^, and 3D^pol^.
